# A case of MCA arising from ICA: a case report

**DOI:** 10.1186/s40792-024-01886-x

**Published:** 2024-04-15

**Authors:** Kai Goyo, Kei Ishimaru, Taichi Miyaji, Masumi Takamoto, Noriaki Kashu, Katsuya Watanabe, Kenji Takagi, Kyosuke Habu, Yusuke Ogi, Hironori Matsumoto, Satoshi Kikuchi, Hiroki Sugishita, Satoshi Akita, Motohira Yoshida, Shigehiro Koga, Taro Oshikiri

**Affiliations:** 1 Department of Gastrointestinal Surgery and Surgical Oncology, Ehime University Graduate School of Medicine, 454 Shitsukawa, Toon, Ehime 791-0295 Japan; 2 Department of Minimally Invasive Gastroenterology, Ehime University Graduate School of Medicine, Toon, Japan

**Keywords:** ICA, MCA, ICG fluorescence imaging

## Abstract

**Background:**

Complete mesocolic excision (CME) and central vascular detachment are very important procedures in surgery for colorectal cancer. Preoperative and intraoperative assessments of the anatomy of major colorectal vessels are necessary to avoid massive bleeding, especially in endoscopic surgery. A case with a rare anomaly in which the middle colic artery (MCA) and ileocolic artery (ICA) had a common trunk is reported.

**Case presentation:**

The patient was a 73-year-old woman diagnosed with ascending colon cancer on colonoscopy. Preoperative abdominal contrast-enhanced computed tomography confirmed that the MCA and ICA had a common trunk. She underwent laparoscopic ileocecal resection for the ascending colon cancer with D3 lymph node dissection. Intraoperative indocyanine green fluorescence imaging was conducted. After confirming vessel bifurcation, the ICA was dissected at the distal end of the MCA bifurcation. The patient has been followed as an outpatient, with no signs of recurrence as of 2 years postoperatively.

**Conclusion:**

A case of an ascending colon cancer with a unique vascular bifurcation pattern was presented. Preoperative and intraoperative evaluations of the major colorectal vessels are very important for preventing perioperative and postoperative complications.

## Background

The promotion and application of complete mesocolic excision (CME) have enormous benefits for patients with colon cancer. In 2009, Hohenberger proposed the concept of CME and central vessel ligation, which emphasizes the importance of vascular root ligation and complete lymph node dissection [1]. Anatomic variations of the right colon vasculature are complex [2], and the improper management of vessels during laparoscopic surgery can cause vascular complications [3]. A case of a rare vascular bifurcation in which the middle colic artery (MCA) and ileocolic artery (ICA) had a common trunk is presented. In this case, preoperative computed tomography (CT) and intraoperative indocyanine green fluorescence imaging enabled safe laparoscopic ileocecal resection and D3 lymph node dissection to be performed. Preoperative and intraoperative vascular evaluations are important for conducting surgery with no complications.

## Case presentation

**Fig. 1:**
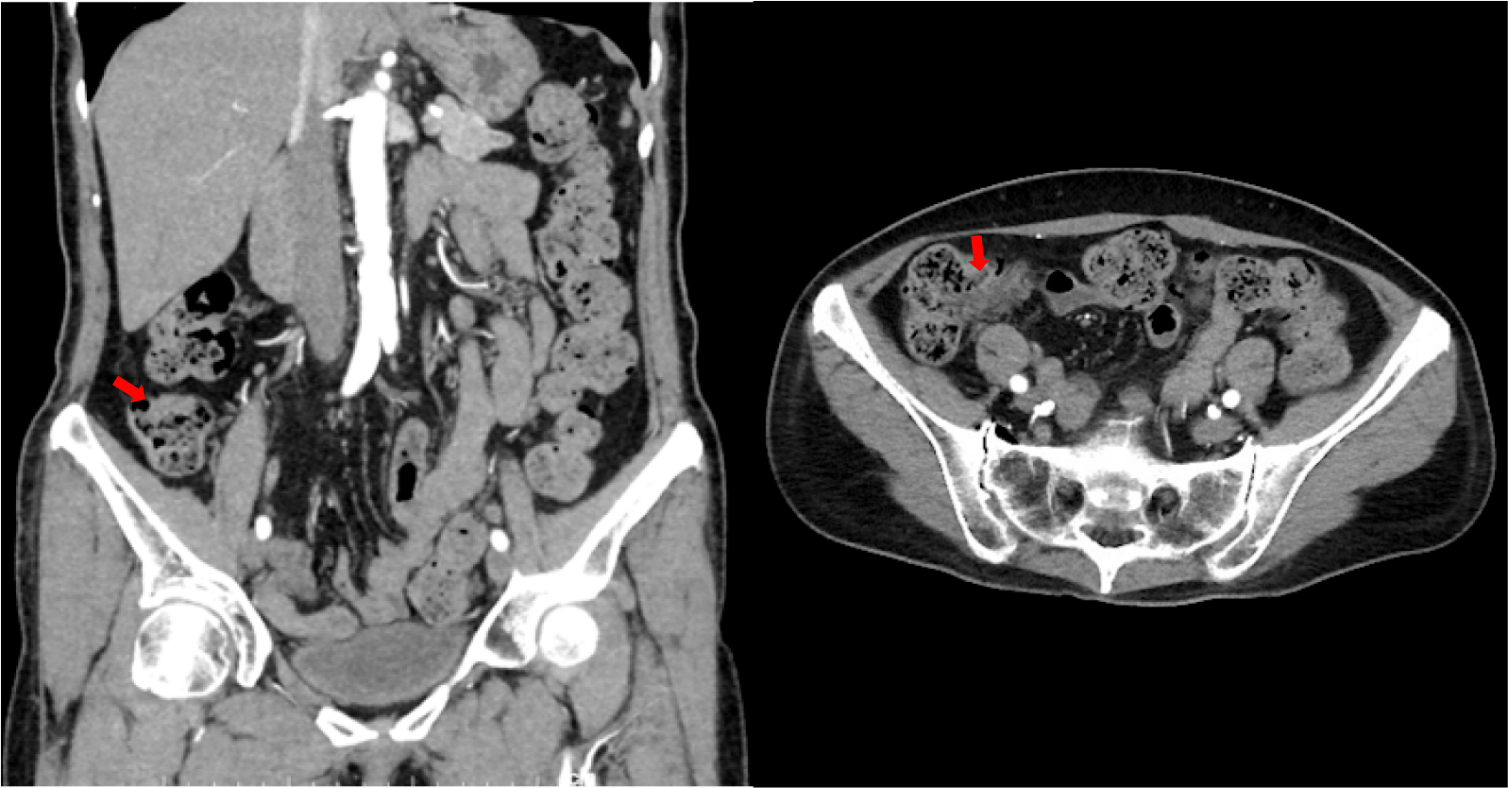
Abdominal contrast-enhanced CT images. CT revealed wall thickness at the ascending colon (red arrow)

**Fig. 2:**
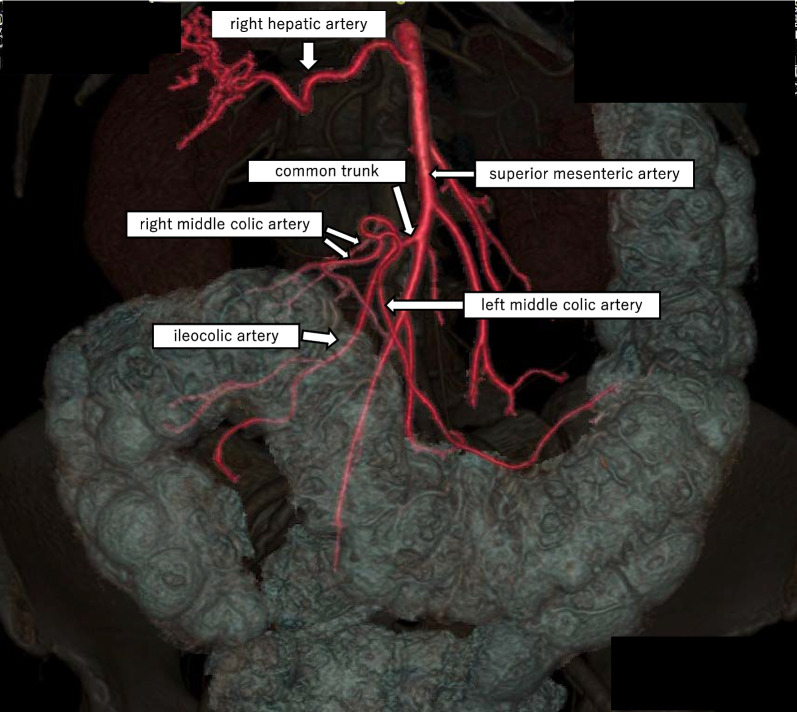
3D-CTA image (artery). 3D-CTA confirmed that the MCA and ICA had a common trunk, and an absent of the RCA

**Fig. 3:**
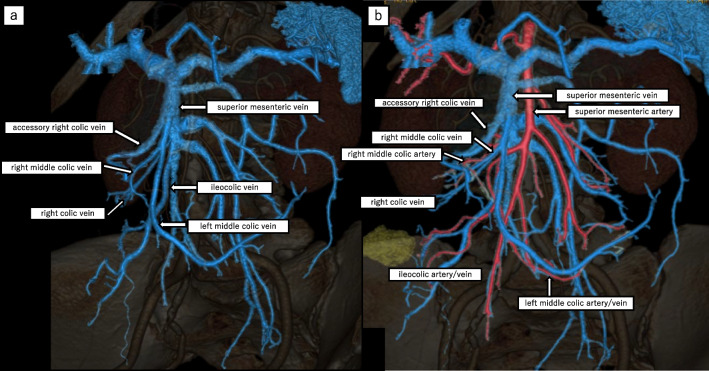
3D-CTA image (**a** vein, **b** artery and vein). **a** 3D-CTA confirmed that the RCV, MCV and ICV arise from SMV. **b** The relationship between veins and arteries

The patient was a 73-year-old woman who was referred to another institution due to bloody stool. On colonoscopy revealed a Type 2 mass at the ascending colon, 5 cm distal from the ileocecal valve, was seen. Biopsy suggested moderately differentiated adenocarcinoma. CT showed wall thickening at the ascending colon (Fig. 1). There was no apparent lymph node metastasis or remote metastasis. Three-dimensional CT angiography (3D-CTA) confirmed that the MCA and ICA had a common trunk, the right hepatic artery originated from the superior mesenteric artery (SMA), and right colic artery (RCA) was absent (Fig. 2). The Inferior mesenteric artery arose from the aorta as usual, and the accessory MCA is absent. The right colic vein (RCV), middle colic vein (MCV), and iliocolic vein (ICV) each arose from the superior mesenteric vein (SMV) respectively (Fig. 3). Serum carcinoembryonic antigen and the carbohydrate antigen 19–9 serum levels were within the normal ranges. The preoperative diagnosis was cT3N0M0 (Japanese Classification Colorectal Carcinoma, 9th Edition) ascending colon cancer. The patient underwent laparoscopic ileocecal resection with D3 lymph node dissection for ascending colon cancer. The operation time was 316 min with minimal bleeding.

**Fig. 4:**
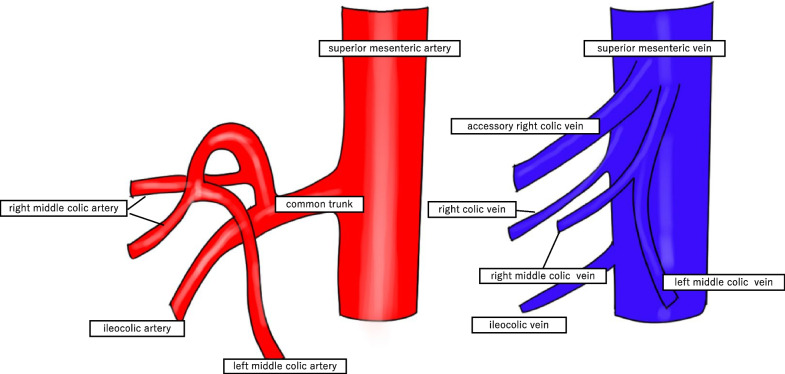
Schema of the arterial and venous runs

**Fig. 5:**
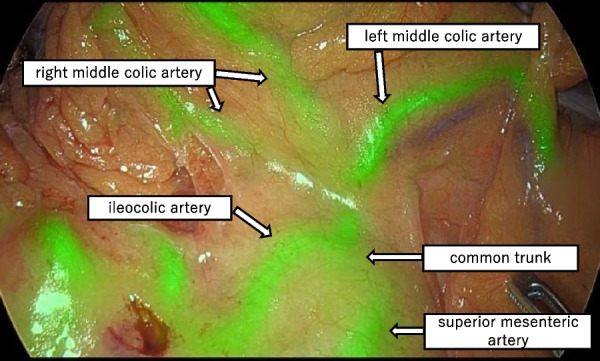
Intraoperative ICG fluorescence imaging. Intraoperative ICG fluorescence imaging showed the MCA originate from the ICA

**Fig. 6:**
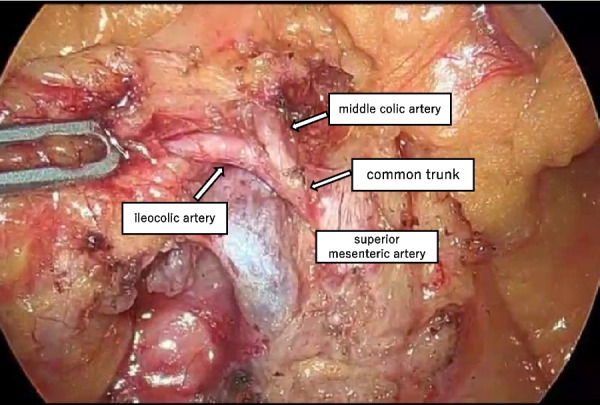
Intraoperative findings. Intraoperative findings, after confirmed the artery vasculature, we removed the ICA at the distal end of the bifurcation of the MCA

During the laparoscopy, a 1688 AIM 4K Camera® (Stryker [Kalamazoo, MI, USA]) was used. A total of 2 mL of the ICG dye was injected, and the camera mode was switched to the overlay function. Thus, the MCA was confirmed to originate from the common trunk with the ICA, as seen on the preoperative examination (Figs. 4 and 5). The ICA was dissected at the distal end of the bifurcation of the MCA (Fig. 6). Based on the resected specimens, the ascending colon cancer classification was pT3N0M0 Stage IIa (Japanese Classification Colorectal Carcinoma, 9th Edition). The patient continues to receive outpatient follow-up care, with no signs of recurrence as of 2 years postoperatively.

## Discussion

In colon cancer surgery, CME, which involves ligation and dissection of the major colorectal vessels at the root and en bloc resection of the mesentery, is a standard technique. CME is required to reduce the risk of recurrence and to improve long-term outcomes. CME is important for reducing the risk of local recurrence and unexpected bleeding after colon cancer surgery. However, proper artery ligation of the right-side colon may be difficult due to variations in arterial vasculature. A previous study examined the occurrence of vascular variations in patients undergoing radical right hemicolectomy and noted that a failure to detect such variations may cause unexpected bleeding [4]. Therefore, it is important to confirm any vascular variations in the right colon. The present case had a rare vascular variation in which the MCA and ICA had a common trunk. Sharing this discovery will raise awareness of such variations and aid surgeons when conducting right colon cancer surgery.

Most reports of variations in the right-sided colic artery dealt with the origin and defects of each branch. In a meta-analysis of 4,691 cases by Robert et al [6], the ICA, RCA and MCA were present in 99.7% (95% CI 99.4%–99.8%), 72.6% (95% CI 61.3%–82.5%) and 96.9% (95% CI 94.2%–98.8%) of patients, respectively. The RCA shared a common trunk with the ICA and MCA in 13.2% and 17.7% of patients, respectively. In the present case, the MCA and ICA were present, but the RCA was absent.

Gamo et al [7] examined 50 cadavers and 560 CTs, and propose that the SMA has four branching patterns (patterns I to IV): Pattern I, the MCA, RCA, and ICA arise separately from the SMA; Pattern II, the three right branches of the SMA arise from a common trunk; Pattern IIa, the MCA and RCA arise from a common trunk; Pattern IIb, the ICA and RCA arise from a common trunk; Pattern IIc, the three arteries arise from a common trunk; Pattern III, the RCA is not present; and Pattern IV, the RCA is found as an accessory branch. Using this classification, the present case conformed to Pattern IIc, which accounted for 2 of 560 (0.35%) cases in their study. The cases of the MCA having a common trunk with the ICA [7–9] are summarized in Table 1; seven cases, including the present case, have been reported, in which the RCA was absent in three.

**Table 1.** Cases of the MCA have a common trunk with the ICA

Cases of the MCA have a common trunk with the ICA			
Year	Author	Common trunk	Number
2016	Gamo7)	MCA, RCA, ICA	2
2021	Mukai8)	MCA, RCA, ICA	1
2022	Stepan9)	MCA, ICA (RCA is absent)	3
2024	our case	MCA, ICA (RCA is absent)	1

The MCA supplies blood to the transverse colon and typically (> 97% of cases) arises from the SMA [5, 10]. An MCA origin anomaly is extremely rare, and few cases have been reported in the literature [10–15] (Table 2).

**Table 2.** Cases of MCA origin anomaly

Cases of MCA origin anomaly		
Year	Author	Origin of MCA
1989	Makomasa11)	Dorsal pancreatic artery
2004	Yídírím12)	Coeliac trunk
2019	Kwong13)	Gastroduodenal artery
2022	Milnerowicz10)	Aorta
2022	Murono14)	Aorta
2024	Maeda15)	Splenic artery

If the anatomy of the vasculature is unknown preoperatively, lymphadenectomy may be inadequate, and bleeding may occur due to vascular injury. Recently, with the widespread use of multi-slice CT for high-resolution imaging using a slice thickness of 1 mm along with image construction software, constructing vascular images and obtaining preoperative information on the arterial bifurcation pattern have become easier. Preoperative 3D-CT reportedly has high sensitivity, specificity, accuracy, and reliability in establishing mesenteric vessel anatomy. Nonetheless, false-negative and false-positive CT findings do occur. Nesgaard et al. [16] reported a diagnostic accuracy of 97.1%, sensitivity of 85.7%, and specificity of 95.2% for preoperative CT image reconstruction of surgical findings or resected specimens. Intraoperative confirmation may be necessary, since preoperative imaging may be inaccurate, particularly in patients with a higher body mass index. In the present case, preoperative multi-slice CT and intraoperative ICG fluorescence imaging with the overlay function (for confirmation of the vascular branching observed on CT) were conducted to perform the surgery without complications.

ICG fluorescence imaging is widely used in gastrointestinal surgery and is considered useful for reducing anastomotic leakage [17]. In the present case, ICG fluorescence imaging was used to identify the artery, not to evaluate the colonic blood flow. PubMed was searched using “ICG”, “intraoperative”, and “artery” as keywords, and no reports of intraoperative ICG examinations to confirm the courses of the colonic vessels were found. In the present case, the colic arteries were clearly visualized with ICG fluorescence imaging; therefore, this method would be useful for determining the optimal vessel resection, especially with abnormal variation of vessels.

## Conclusion

A case of an ascending colon cancer with a unique vascular bifurcation pattern was described. Preoperative and intraoperative evaluations of the right colon vasculature are very important for preventing complications.

## Data Availability

Data sharing is not applicable to this article as no new data were created or analyzed in this study.

## Abbreviations

CME: Complete mesocolic excision
MCA: Middle colic artery
ICA: Ileocolic artery
CT: Computed tomography
3D-CT: Three-dimensional CT angiography
SMA: Superior mesenteric artery
RCA: Right colic artery
RCV: Right coli vein
MCV: Middle colic vein
ICV: Ileocolic vein
SMV: Superior mesenteric vein
